# Developing an accurate empirical correlation for predicting anti-cancer drugs’ dissolution in supercritical carbon dioxide

**DOI:** 10.1038/s41598-022-13233-x

**Published:** 2022-06-07

**Authors:** Fardad Faress, Amin Yari, Fereshteh Rajabi Kouchi, Ava Safari Nezhad, Alireza Hadizadeh, Leili Sharif Bakhtiar, Yousef Naserzadeh, Niloufar Mahmoudi

**Affiliations:** 1 Department of Business, Data Analysis, The University of Texas Rio Grande Valley (UTRGV), Edinburg, TX 78539 USA; 2 Faculty of Pharmacy, Isfahan University of Medical Sciences (IUMS), Isfahan, Iran; 3 Micron School of Materials Science and Engineering, Boise State University, Boise, ID 83725 USA; 4 Faculty of Pharmacy, Bogomolets National Medical University, Kyiv, Ukraine; 5 MD, School of Medicine, Tehran University of Medical Sciences, Tehran, Iran; 6 Research Center for Advanced Technologies in Cardiovascular Medicine, Cardiovascular Diseases Research Center Institute, Tehran University of Medical Sciences, Tehran, Iran; 7 Protein Research Center, Shahid Beheshti University, Tehran, Iran; 8 Department of AgroBiotechnology, Peoples’ Friendship University of Russia (RUDN University), 117198 Moscow, Russia

**Keywords:** Drug discovery, Chemistry, Chemical engineering

## Abstract

This study introduces a universal correlation based on the modified version of the Arrhenius equation to estimate the solubility of anti-cancer drugs in supercritical carbon dioxide (CO_2_). A combination of an Arrhenius-shape term and a departure function was proposed to estimate the solubility of anti-cancer drugs in supercritical CO_2_. This modified Arrhenius correlation predicts the solubility of anti-cancer drugs in supercritical CO_2_ from pressure, temperature, and carbon dioxide density. The pre-exponential of the Arrhenius linearly relates to the temperature and carbon dioxide density, and its exponential term is an inverse function of pressure. Moreover, the departure function linearly correlates with the natural logarithm of the ratio of carbon dioxide density to the temperature. The reliability of the proposed correlation is validated using all literature data for solubility of anti-cancer drugs in supercritical CO_2_. Furthermore, the predictive performance of the modified Arrhenius correlation is compared with ten available empirical correlations in the literature. Our developed correlation presents the absolute average relative deviation (AARD) of 9.54% for predicting 316 experimental measurements. On the other hand, the most accurate correlation in the literature presents the AARD = 14.90% over the same database. Indeed, 56.2% accuracy improvement in the solubility prediction of the anti-cancer drugs in supercritical CO_2_ is the primary outcome of the current study.

## Introduction

Supercritical is a technical phrase to refer to operating conditions where both pressure and temperature are higher than their critical values for a given substance^1^. It is widely accepted that supercritical fluids (SCF) pose some valuable advantages over traditional solvents (liquid-like density, gas-like transport properties, low surface tension, and good mass transfer capacity)^2^. These characteristics have drawn attention to the SCFs as solvent media for supercritical extraction/purification purposes in a wide range of applications^1^. Carbon dioxide (CO_2_) is likely the most trustful supercritical fluid in energy^3^, food^4^, pharmaceutical^5,6^, and bioactive agent delivery^7–10^ applications. Indeed, the non-toxic, inflammable, and non-explosive nature of supercritical carbon dioxide (SCCO_2_) is responsible for these trustful applications^2^. Furthermore, the SCCO_2_ critical characteristics are mild (temperature = 31.1 °C, pressure = 73.8 bar)^11^, it is recyclable, simply available at low expense, and covers the real-field requirement^2^.

The SCCO_2_ has outstanding applications in pharmaceutical manufacturing processes^12,13^. Drug solubility in SCF is the most crucial information for the feasibility study, development, and construction of the pharmaceutical processes utilized the supercritical fluids as solvent media^14^. Since cancer is a leading cause of human death all around the world^15–18^, researchers experimentally measured the solubility of different anti-cancer drugs in supercritical CO_2_, including sorafenib tosylate^19^, sunitinib malate^20^, azathioprine^21^, busulfan^22^, tamoxifen^23^, letrozole^24^, tamsulosin^25^, capecitabine^26^, paclitaxel^27^, 5-fluorouracil^27^, thymidine^27^, and decitabine^28^. Unfortunately, the laboratory measurement of drug solubility in supercritical CO_2_ at whole ranges of pressures and temperatures is time-consuming and requires high economic expenses^2^.

In order to resolve these operating and economic problems, different thermodynamic-based models (known as the equation of state)^29–32^, intelligent paradigms^14^, predictive model^33,34^, and empirical correlations^35–44^ are proposed to simulate different phenomena, including estimating solids solubility in SCCO_2_. Sodeifian et al. compared the accuracy of the Peng-Robinson (PR), Soave–Redlich–Kwong (SRK), and available empirical correlations for predicting solubility of sorafenib tosylate^19^, sunitinib malate^20^, and azathioprine^21^ anti-cancer drugs in SCCO_2_. Performances of the PR equation of state, statistical associating fluid theory of variable range (SAFT-VR), and six empirical correlations for predicting tamsulosin solubility in supercritical CO_2_ have also been compared^25^. Generally, the estimation methods of drug solubility in the SCCO_2_ using the equations of state (EoS) are often mathematically complicated^2^, require high computations efforts^2^, need relatively high entry information^2,45,46^, provide high levels of uncertainty^19^, and may sometimes fail^20^. More precisely, they need the operating conditions, critical properties, and also drug characteristics to deliver their predictions^19,20^.

The least-squares support vector machines^14^, artificial neural networks^47–50^, quantitative structure–property relationships^51^, adaptive neuro-fuzzy inference systems^52,53^, wavelet transform^54–57^, and dynamic simulation^58–60^ are some of the approaches may be used for estimating the solid solubility in supercritical carbon dioxide. Utilizing these intelligent paradigms is only possible when their structure, adjusted hyper-parameters, and performed pre-processing and post-processing stages be completely available^61–65^. Despite an acceptable accuracy of these intelligent methods, some parts of their information are often missed to present, and it is hard or even impossible to be used by other researchers.

The empirical correlations that only need temperature, pressure, and pure SCCO_2_ density to predict solid solubility in supercritical carbon dioxide^29–32^ have attracted greater attention in this regard. In order to escape an unnecessary repetition, the mathematical expressions of these empirical correlations will be reviewed in the subsequent sections (see “Most widely used correlations for drug solubility in SCCO_2_”). The mathematical formulations of these empirical correlations are simple, understandable, ready to use, and their accuracy is often far better than the thermodynamic-based models^19,20^. Moreover, it is possible to incorporate them in an appropriate optimization algorithm to determine the operating condition that maximizes the drug solubility in SCCO_2_.

The current research briefly reviewed ten well-known and reliable empirical correlations for estimating solid solubility in supercritical CO_2_^35–44^. After that, a universal approach based on the modified Arrhenius model is introduced to relate the anti-cancer drug solubility in SCCO_2_. This universal approach added a departure function to the Arrhenius-shape term to estimate the anti-cancer drug solubility in SCCO_2_. The predictive performance of the modified Arrhenius model and available correlations in the literature is compared using all available experimental data for solubility of anti-cancer drugs in SCCO_2_. 316 experimental data for solubility of sorafenib tosylate^19^, sunitinib malate^20^, azathioprine^21^, busulfan^22^, tamoxifen^23^, letrozole^24^, tamsulosin^25^, capecitabine^26^, paclitaxel^27^, 5-fluorouracil^27^, thymidine^27^, and decitabine^28^ in SCCO_2_ are used to perform this comparison. The results show that the modified Arrhenius model improves the previously achieved accuracy in the literature by more than 56.2%.

## Materials and methods

The first part of this section presents the available experimental measurements for the solubility of anti-cancer drugs in supercritical CO_2_. The second part reviews the most well-known empirical models for correlating the solid solubility in SCCO_2_ to the independent variables (pressure, temperature, and pure supercritical CO_2_ density).

### Anti-cancer drugs

As mentioned earlier, cancer is approved as the leading cause of human death worldwide^15^. Therefore, all aspects of anti-cancer drugs, including their solubility in the supercritical CO_2_ are an exciting research topic for both academic and manufacturing purposes. Based on our best knowledge, the solubility of only twelve anti-cancer drugs in the supercritical carbon dioxide were measured up to now. These anti-cancer drugs are sorafenib tosylate^19^, sunitinib malate^20^, azathioprine^21^, busulfan^22^, tamoxifen^23^, letrozole^24^, tamsulosin^25^, capecitabine^26^, paclitaxel^27^, 5-fluorouracil^27^, thymidine^27^, and decitabine^28^. Table 1 separately reports the range of pressure, temperature, supercritical CO_2_ density, and anti-cancer drug solubility for all the laboratory-scale studies. Furthermore, the numbers of available measurements in each research are also shown in this table.

**Table 1:** Literature data for solubility of anti-cancer drugs in supercritical carbon dioxide.

CO_2_ (1) + drug (2)	Temperature (K)	Pressure (MPa)	CO_2_ density (kg/m^3^)	Drug solubility* × 10^6^	No. data
Sorafenib tosylate^19^	308–338	12–27	388–914	0.68–12.57	24
Sunitinib malate^20^	308–338	12–27	388–914	5–85.6	24
Azathioprine^21^	308–338	12–27	388–914	2.7–18.3	24
Busulfan^22^	308–338	12–40	383–971	32.7–865	32
Tamoxifen^23^	308–338	12–40	383–971	18.8–989	32
Letrozole^24^	318–348	12–36	319–922	1.6–85.1	20
Tamsulosin^25^	308–338	12–27	384–914	0.18–10.13	24
Capecitabine^26^	308–348	15.2–35.4	477–955	2.7–158.8	40
Paclitaxel^27^	308–328	10–27.5	654–915	1.2–6.2	21
5-Fluorouracil^27^	308–328	12.5–25	541–901	3.8–14.6	18
Thymidine^27^	308–328	10–30	325–928	1.2–8	25
Decitabine^28^	308–338	12–40	383–971	28.4–1070	32

### Most widely used correlations for drug solubility in SCCO_2_

The developed empirical correlations by Chrastil^35^, Jouyban et al.^36^, Kumar and Johnstone^37^, Garlapati and Madras^38^, Bian et al.^39^, Bartle et al.^40^, Méndez-Santiago and Teja^41^, Sodeifian et al.^42^, Tan et al.^43^, and Gordillo et al.^44^ are widely used to estimate drug solubility in supercritical carbon dioxide. It should be mentioned that some of these correlations were initially proposed for the prediction of the solid (not specifically drug) solubility in SCCO_2_. However, researchers preserved their mathematical forms, readjusted their coefficients, and modified them to be applied in the drug/SCCO_2_ phase equilibria modeling^19,25,26,28^.

The mathematical formulations of these empirical correlations are given in Table 2. It should be mentioned that excluding Eq. (1) that predicts the solubility in terms of the mass of solids per volume of the solvent ($c_{2}$), all other considered correlations provide the solubility in terms of mole fraction unit ($y_{2}$). Furthermore, temperature, pressure, and pure SCCO_2_ density are designated by $T$, $P$, and $\rho$, respectively. Finally, the coefficients of the correlations are shown by the $a_{1}$ to $a_{6}$ notations.

**Table 2:** Available empirical correlations for solute/drug solubility in supercritical carbon dioxide.

Correlation	Formula	
Chrastil^35^	$c_{2} = \rho^{{a\,_{1} \,}} \;\exp \;\left( {\frac{{a_{2} }}{T} + a_{3} } \right)$	Equation (1)
Jouyban et al.^36^	$\ln \;\left( {y_{2} } \right) = a_{1} + a_{2} \;\rho + a_{3} \;P^{2} + a_{4} \;P\;T + a_{5} \,\frac{T}{P} + a_{6} \;\ln \;\left( \rho \right)$	Equation (2)
Kumar and Johnstone^37^	$\ln \,\left( {y_{2} } \right) = a_{1} + a_{2} \;\rho + \frac{{a_{3} }}{T}$	Equation (3)
Garlapati and Madras^38^	$\,\ln \,\left( {y_{2} } \right)\, = \,a_{1} \, + \,\left( {a_{2} \, + \,a_{3} \,\rho } \right)\,\ln \left( \rho \right)\, + \,\frac{{a_{4} }}{T}\, + \,a_{5} \,\ln \,\left( {\rho T} \right)$	Equation (4)
Bian et al.^39^	$\,y_{2} \, = \,\rho^{{\left( {a\,_{1} \, + \,a_{\,2} \,\rho } \right)}} \,\exp \left( {\frac{{a_{3} }}{T}\, + \,\frac{{a_{4} \,\rho \,}}{T}\, + \,a_{5} } \right)$	Equation (5)
Bartle et al.^40^	$\ln \,\left( {\frac{{y_{2} \,P}}{{P_{ref} }}} \right)\, = \,a_{1} \, + \,\frac{{a_{2} }}{T}\, + \,a_{3} \,\left( {\rho \, - \,\rho_{ref} } \right)\,,\,\,\,P_{ref} \, = \,0.1MPa,\,\rho_{ref} \, = \,700\,kg/m^{3} \,\,\,\,\,\,$	Equation (6)
Méndez-Santiago and Teja^41^	$T\;\ln \;\left( {y_{2} \;P} \right) = a_{1} + a_{2} \;\rho + a_{3} \;T$	Equation (7)
Sodeifian et al.^42^	$\ln \;\left( {y_{2} } \right) = a_{1} + a_{2} \;\frac{{P^{2} }}{T} + a_{3} \;\ln \left( {\rho T} \right) + a_{4} \;\rho \ln \left( \rho \right) + a_{5} \;P\;\ln \;\left( T \right) + a_{6} \;\frac{\ln \;\left( \rho \right)}{T}$	Equation (8)
Tan et al.^43^	$\ln \;\left( {y_{2} } \right) = a_{1} \;\ln \;\left( {\rho T} \right) + a_{2} \;\rho + \frac{{a_{3} }}{T} + a_{4}$	Equation (9)
Gordillo et al.^44^	$\ln \;\left( {y_{2} } \right) = a_{1} + a_{2} \;P + a_{3} \;P^{2} + a_{4} \,P\;T + a_{5} \;T + a_{6} \;T^{2}$	Equation (10)

Excluding the pure SCCO_2_ density of the Eq. (3) that is in the kmol/m^3^ unit, the units of all other variables are in complete agreement with those unites presented in Table 1.

## Results and discussion

This section presents the idea of developing the modified Arrhenius correlation, adjusts its unknown coefficients, and compares its accuracy with other available correlations. The next part of this section is devoted to the performance analysis of the modified Arrhenius correlation using different graphical methods. Finally, the modified Arrhenius correlation is employed to monitor the effect of operating conditions on the anti-cancer drug solubility in SCCO_2_.

### Developing the modified Arrhenius correlation

The massive data processing stages are performed on the experimental values of solubility of each drug in SCCO_2_ to reach a general form of the proposed correlation as follows:

11 $$y_{2} = Arrhenius\;term \, + \, departure\;function$$

Equation (11) states that the anti-cancer drug solubility in the SCCO_2_ can be accurately estimated by combining an Arrhenius term and a departure function.

At this stage, it is necessary to clarify how the pre-exponential and exponential parts of the Arrhenius term are related to the influential variables. Then, the departure function incorporates to reduce the deviation between the Arrhenius term predictions and experimental measurements.

Spearman and Pearson are two well-known relevancy discovery scenarios in the field of data processing^62^. They introduce the relevancy between a pair of feature-response variables by a factor in the range of − 1 to + 1. The minus, zero, and positive factors correspond with indirect dependency, no-relation, and direct dependency, respectively^62,66^. The strength of either direct or indirect relevancy increases by increasing the magnitude of factors^67^. Furthermore, the higher absolute value of the Spearman than the Pearson factor confirms that the non-linear relationship is stronger than the linear one and vice versa^62,66^.

**Figure 1:**
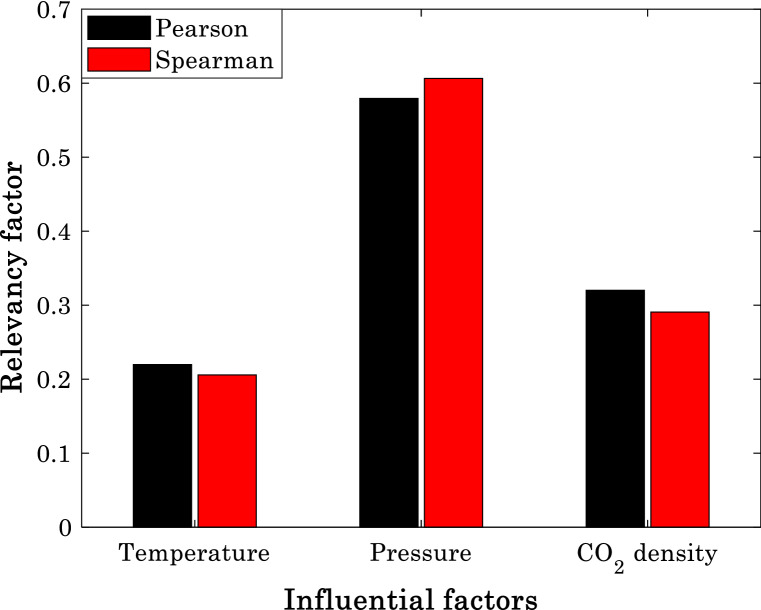
Relevancy between the solubility of anti-cancer drugs in supercritical CO_2_ and temperature, pressure, and carbon dioxide density.

Figure 1 exhibits the values of relevancy factor between anti-cancer drug solubility and pressure, temperature, and pure SCCO_2_ density. This figure confirms that direct relationships exist between the response and all feature variables. The anti-cancer drug solubility has the strongest relationship with the pressure and weakest dependency to the temperature. Moreover, since the Pearson factors for temperature and CO_2_ density are higher than the Spearman ones, the linear relationship is superior to the non-linear one. The higher Spearman factor than Pearson for the pressure shows that the anti-cancer drug solubility non-linearly relates to the pressure.

These findings are in complete agreement with the mathematical form of the Arrhenius model. Indeed, the pre-exponential term can be a function of temperature and CO_2_ density, and the exponential term provides the non-linear relation with the pressure.

**Figure 2:**
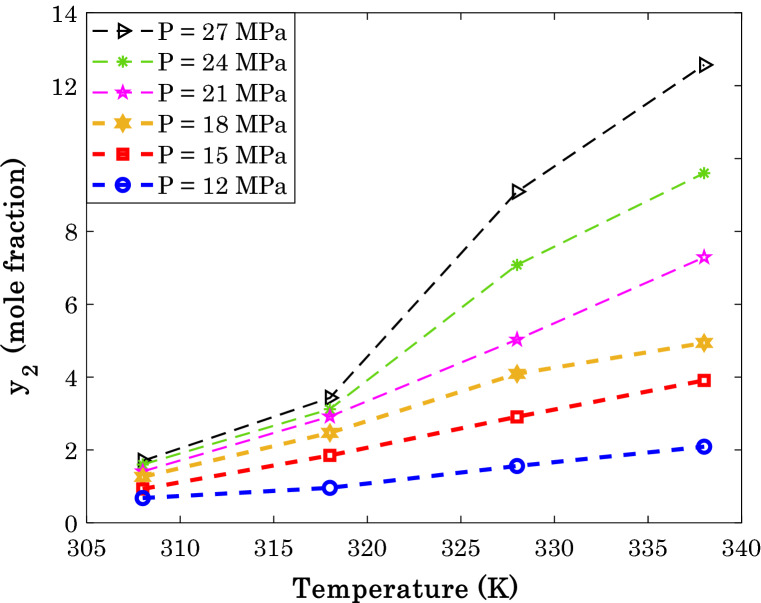
Dependency of sorafenib tosylate solubility in the supercritical CO_2_ on the isobaric variation of temperature (the cartesian coordinate).

**Figure 3:**
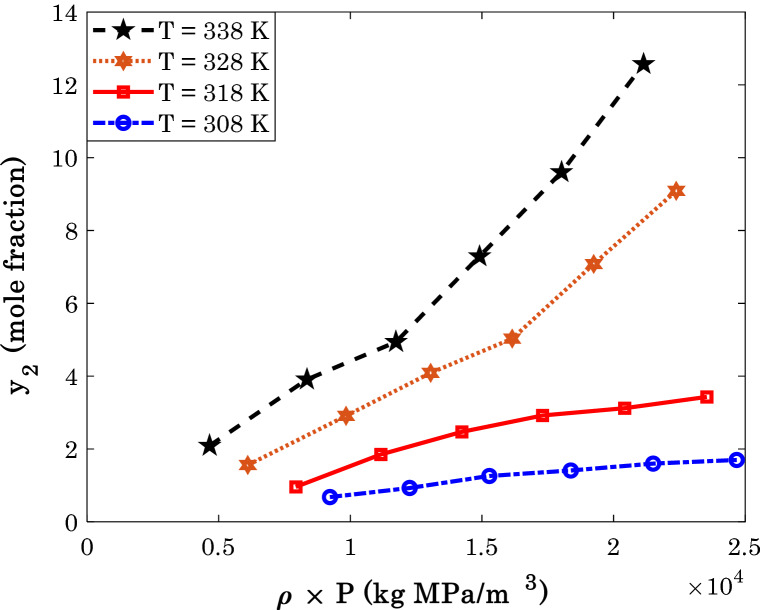
The variation of sorafenib tosylate solubility in the SCCO_2_ by the solvent density (the cartesian coordinate).

**Figure 4:**
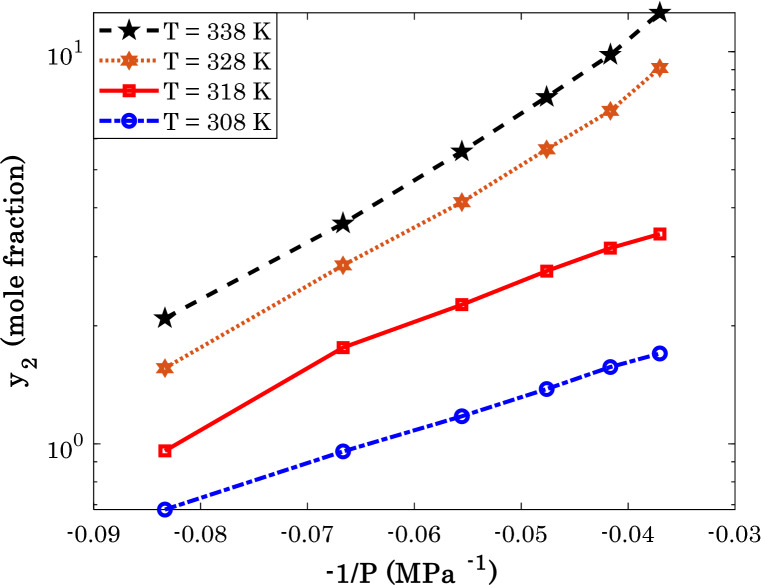
The effect of the inverse pressure on the sorafenib tosylate solubility in the SCCO_2_ (the semi-logarithm coordinate).

The previous findings specify the linear dependency of the anti-cancer drug solubility on temperature and CO_2_ density and its non-linear relationship with the pressure. Figures 2, 3 and 4 are plotted to approve these findings through visual inspection.

The experimental values of typical anti-cancer drug solubility in the SCCO_2_ as a function of temperature are shown in Fig. 2. This figure approves that the temperature dependency of the solubility of the anti-cancer drugs is almost linear. The departure function is efficiently involved in compensating for the deviation from the linear relationship.

Since the density of the pure SCCO_2_ changes by both pressure and temperature, it is impossible to monitor the dependency of the anti-cancer drug solubility on the CO_2_ density in the two-dimensional graph. Hence, Fig. 3 depicts the solubility of a typical anti-cancer drug versus the product of pressure and CO_2_ density. The linear dependency of the anti-cancer drug solubility on the pure SCCO_2_ density can be inferred from this figure. Similar to the temperature, the departure function can compensate for the deviation from the linear relationship between drug solubility and CO_2_ density.

The semi-logarithm presentation of typical anti-cancer drug solubility in the SCCO_2_ versus the inverse of pressure is shown in Fig. 4. This figure approves that the anti-cancer drug solubility in SCCO_2_ exponentially relates to the inverse of pressure, i.e., $\exp \,\left( { - \,E_{a} /P} \right)$. The observed deviation between the exponential data and predictions of the Arrhenius term for the pressure effect is then reduced by applying the departure function.

In summary, the following Arrhenius-shape correlation^68^ is inferred to estimate the anti-cancer drug solubility in the SCCO_2_ (Eq. 12).

12 $$Arrhenius\;term = f_{1} \left( {T,\;\rho } \right)\;\exp \;\left( { - \;\frac{{E_{a} }}{P}} \right)$$

It is expected that some deviations observe between the Arrhenius term predictions and actual solubility data. However, it is possible to enhance the accuracy of the Arrhenius-shape model by diminishing the observed deviations. Therefore, a new term (i.e., departure function) adds to the Arrhenius-shape part to compensate for this deviation. The observed deviation shows the highest compatibility with the natural logarithm of the CO_2_ density to the temperature as follows:

13 $$departure \, function = \,\,f_{2} \left( {\ln \left( {\frac{\rho }{T}} \right)\,} \right)\,$$

In summary, the general form of the proposed correlation achieves by combining the Arrhenius term and departure function (Eq. 14).

14 $$y_{2} = f_{1} \left( {T,\;\rho } \right)\;\exp \;\left( { - \;\frac{{E_{a} }}{P}} \right) + f_{2} \left( {\ln \left( {\frac{\rho }{T}} \right)} \right)$$

Equation (15) presents the final form of the proposed correlation for estimating the solubility of the anti-cancer drugs in supercritical CO_2_.

15 $$y_{2} = \left( {a_{1} \;T + a_{2} \;\rho + a_{3} } \right)\;\exp \;\left( { - \,\frac{{a_{4} }}{P}} \right) + a_{5} \;\ln \left( {\frac{\rho }{T}} \right) + a_{6}$$

The pre-exponential part of the Arrhenius term linearly combines the effect of temperature and CO_2_ density, while its exponential part is a function of pressure only. The departure function linearly relates to the natural logarithm of the CO_2_ density to the temperature ratio.

#### Adjusting the coefficients of the correlations

After determining the general form of the proposed correlation, it is now necessary to adjust its coefficients using an appropriate method. The differential evolution (DE) optimization algorithm^69,70^ is employed to adjust these unknown coefficients through a non-linear regression process. The absolute average relative deviation (AARD%) between the model predictions and actual measurements is an objective function for the optimization stage. The AARD% formula can be expressed by Eq. (16)^71^.

16 $$AARD\% = \frac{100}{N}\;\sum {\left( {\frac{{\left| {y_{2}^{\exp } - y_{2}^{cal} } \right|}}{{y_{2}^{\exp } }}} \right)}_{i} \;\;\;i = 1,\;2,\; \ldots ,\;N$$

Table 3 presents the adjusted coefficients for estimating the solubility of different anti-cancer drugs in the SCCO_2_.

**Table 3:** Adjusted coefficients of the proposed correlation for estimating the solubility of anti-cancer drugs in supercritical CO_2_.

Drug	a_1_ × 10^–6^	a_2_ × 10^–6^	a_3_ × 10^–6^	a_4_	a_5_ × 10^–6^	a_6_ × 10^–6^
Sorafenib tosylate	1.4247605573	− 0.0500529210	− 385.827674360	45.3662839688	0	0.349064452
Sunitinib malate	0.2647256734	− 1.4200164959	1355.66373666	36.2840647550	− 12.8796352	0
Azathioprine	0.9468557792	− 0.0163521099	− 266.098396089	24.1069578946	8.36900373	− 4.83860672
Busulfan	60.280909168	− 16.630567159	− 1344.60675047	78.2721455261	76.7145899	0
Tamoxifen	144.11848954	− 42.703480295	− 1974.01401538	99.6434546809	41.05460792	6.14532671
Letrozole	8.7649418657	− 2.9569956441	4.94014077630	80.8669042062	4.145085191	− 0.53838515
Tamsulosin	0.7923204828	0.1478689559	− 368.263373717	35.1646118112	0	2.51134612
Capecitabine	48.093681561	− 12.750147899	− 2037.79351684	123.522295303	17.1813407	− 8.09385756
Paclitaxel	0.0068347581	0.0253752394	− 6.61523852780	14.6087222619	− 22.8448049	17.3239309
5-Fluorouracil	0.5186887168	− 0.1340640969	− 1.39609503380	44.0728502801	− 10.2690278	11.7076100
Thymidine	0.2122407854	0.0094685158	− 70.3123673279	18.0254848942	− 2.51330781	3.01138517
Decitabine	117.68771002	− 10.092201917	− 25,295.9332530	74.7951605316	48.3284747	0

The literature has already used some correlations (see Table 2) to estimate the anti-cancer drug solubility in SCCO_2_. Therefore, the researchers readjusted coefficients and apply them in the drug/SCCO_2_ systems. However, readjusting the coefficients of other ones are accomplished in the current study. Supplementary file presents the coefficients of the considered correlations for solubility of all anti-cancer drugs in supercritical CO_2_. The optimization algorithm and objective function like that utilized for the modified Arrhenius model are also employed to adjust the coefficients of the literature correlations.

### Comparative analysis

This section compares the uncertainty in the predictions of the modified Arrhenius model and available correlations in the literature for solubility of anti-cancer drugs in SCCO_2_. The prediction uncertainty of all considered empirical correlations is measured in terms of the AARD% and reported in Table 4. First of all, it is better to clarify that the highlighted cells (gray color) are calculated in the present study, and the clean cells are those reported in the literature. As mentioned earlier, the associated coefficients for calculating this AARD% are presented in Supplementary file. The cells shown by the bold font are the smallest AARD% (the best results) obtained for estimating a specific anti-cancer drug in supercritical CO_2_. It is obvious that the modified Arrhenius correlation provides the most accurate results for solubility of six out of twelve anti-cancer drugs in SCCO_2_ (i.e., sorafenib tosylate, sunitinib malate, azathioprine, tamsulosin, 5-fluorouracil, thymidine).

**Table 4:** Uncertainty of the proposed model and available correlations in the literature in terms of AARD% (the italicized cells are calculated in the current study; the bold font values show the most accurate predictions).

Drug	Empirical correlation					
	Modified Arrhenius	Chrastil^35^	Jouyban et al.^36^	Kumar and Johnston^37^	Garlapati and Madras^38^	
Sorafenib tosylate	***7.91***	13.90^19^	14.40^19^	12.70^19^	11.00^19^	
Sunitinib malate	***3.89***	21.26^20^	14.20^20^	*38.85*	17.16^20^	
Azathioprine	***4.29***	9.88^21^	10.21^21^	*16.26*	8.62^21^	
Busulfan	*7.41*	11.20^22^	*88.70*	7.57^22^	11.20^22^	
Tamoxifen	*12.02*	16.50^23^	*96.87*	11.10^23^	16.40^23^	
Letrozole	*13.21*	22.16	21.50^24^	*39.42*	**7.14**^24^	
Tamsulosin	***9.27***	22.11^25^	*82.70*	15.20^25^	*24.91*	
Capecitabine	*11.42*	12.20^26^	11.90^26^	10.30^26^	*43.48*	
Paclitaxel	*9.69*	*28.90*	*80.95*	*38.89*	*11.79*	
5-Fluorouracil	***8.39***	*19.48*	*69.90*	*19.48*	*22.77*	
Thymidine	***16.64***	*25.10*	*91.77*	*29.86*	*32.48*	
Decitabine	*9.11*	15.30^28^	*88.88*	9.04^28^	15.30^28^	
Overall	**9.54**	17.42	56.51	18.97	19.64	

Drug	Empirical correlation					
	Bian et al.^39^	Bartle et al.^40^	MST^41^	Sodeifian et al.^42^	Tan et al.^43^	Gordillo^44^
Sorafenib tosylate	10.30^19^	13.70^19^	15.30^19^	10.10^19^	*52.21*	*95.85*
Sunitinib malate	21.07^20^	26.11^20^	24.68^20^	12.16^20^	*54.69*	*91.67*
Azathioprine	8.40^21^	12.22^21^	10.70^21^	8.04^21^	*13.72*	*84.91*
Busulfan	***6.55***	11.70^22^	10.70^22^	*25.99*	*32.48*	*96.87*
Tamoxifen	***8.84***	16.10^23^	16.00^23^	*58.03*	*45.91*	*96.87*
Letrozole	*10.42*	*46.61*	15.40^24^	*26.66*	*39.20*	*95.00*
Tamsulosin	14.24^25^	17.08^25^	16.98^25^	13.64^25^	*29.07*	*95.83*
Capecitabine	*33.00*	12.80^26^	9.90^26^	41.47^26^	**9.10**^26^	20.50^26^
Paclitaxel	*15.96*	*50.09*	*55.31*	***9.26***	*18.44*	*95.23*
5-Fluorouracil	*15.59*	*35.34*	*31.98*	*13.86*	*25.52*	*82.99*
Thymidine	*19.68*	*46.90*	*40.32*	*22.31*	*32.78*	*96.00*
Decitabine	***8.82***	15.30^28^	13.30^28^	*80.01*	*49.18*	*96.88*
Overall	14.90	23.24	20.01	30.05	33.19	84.66

On the other hand, the derived correlation by Bian et al.^39^ predicts the solubility of busulfan, tamoxifen, and decitabine in supercritical CO_2_ with the highest accuracy. Finally, the Garlapati and Madras^38^, Sodeifian et al.^42^, and Tan et al.^43^ correlations provide the most accurate predictions for only one anti-cancer drug.

**Figure 5:**
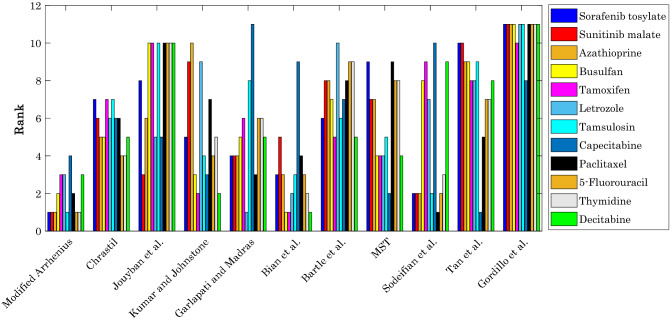
Outcome of the ranking analysis on the accuracy of the developed correlation and those available in the literature.

Figure 5 exhibits the results of ranking analysis on the accuracy of the modified Arrhenius model and available empirical correlations in the literature for calculating the solubility of different anti-cancer drugs in supercritical CO_2_. It can be readily deduced that the proposed correlation in the current study not only presents the most accurate predictions for six anti-cancer drugs, it also has two second and three third ranks. The worst accuracy of the modified Arrhenius correlation is associated with capecitabine solubility in the SCCO_2_ (i.e., the fourth rank). The proposed correlation by Bian et al.^39^ with the three first, two second, four third, one fourth, and one ninth ranks is the next reliable model for the given task. On the other hand, the proposed correlations by Gordillo^44^, Jouyban et al.^36^, and Tan et al.^43^ have the highest levels of uncertainty, respectively.

#### Overall ranking of the correlation

**Figure 6:**
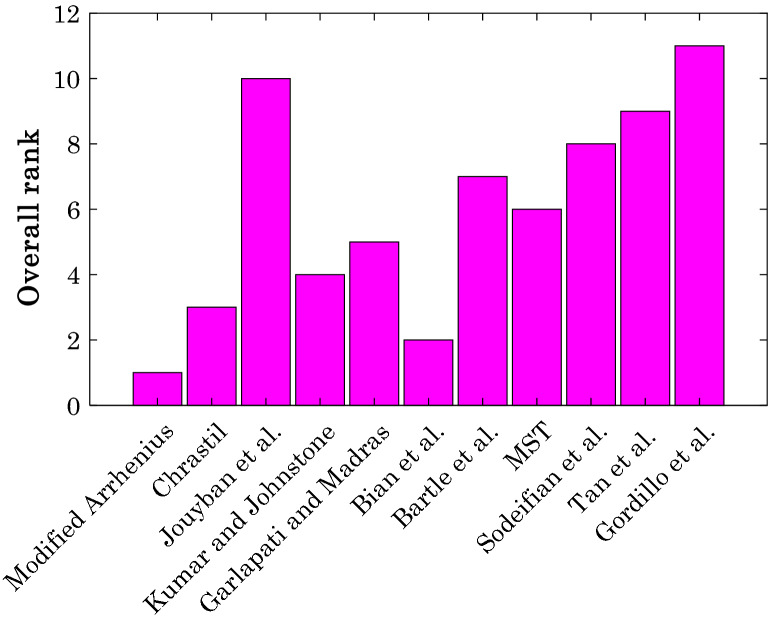
Overall ranking of the considered correlations to predict the solubility of anti-cancer drugs in supercritical carbon dioxide.

This section investigates/compares the accuracy of the modified Arrhenius model and available empirical correlation in the literature for estimating the whole of the database (solubility of all anti-cancer drugs in supercritical CO_2_). Hence, Fig. 6 illustrates the results of ranking analysis for the overall accuracy of the considered empirical correlations.

As expected, the modified Arrhenius correlation (with the smallest overall AARD = 9.54%) takes the first ranking place for the whole of the experimental databank. The Bian et al. correlation^39^ with the overall AARD = 14.90% is the next accurate model for the given purpose. Generally, all available correlations in the literature have the AARD% equal to or higher than 14.9%. Indeed, the modified Arrhenius correlation improves the accuracy of available models in the literature by at least 56.2%.

### Performance monitoring of the modified Arrhenius correlation

**Figure 7:**
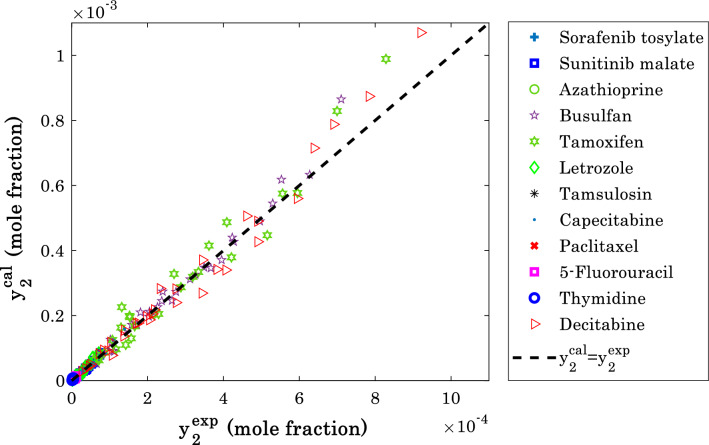
Cross-plot of the modified Arrhenius predictions versus experimental measurements for anti-cancer drug solubility in supercritical CO_2_.

The agreement between the experimental solubility data and calculated values by the developed modified Arrhenius correlation is plotted in Fig. 7. This figure includes the solubility of all anti-cancer drugs in the supercritical carbon dioxide. Despite an infinitesimal range of the solubility data (~ 10^–4^), an acceptable compatibility can be observed between actual and calculated information. The modified Arrhenius correlation provides the R^2^ (regression coefficient, Eq. 17a^72^) of 0.98479 and standard error of 2.02 × 10^–5^ for all 316 experimental data.

17a $$R^{2} = \,1 - \sum\limits_{i = 1}^{N} {\left( {y_{{_{2} }}^{\exp } - y_{{_{2} }}^{cal} } \right)_{i}^{2} /\sum\limits_{i = 1}^{N} {\left( {y_{{_{2} }}^{\exp } - \overline{{y_{{_{2} }}^{\exp } }} } \right)_{i}^{2} } } \,$$

**Figure 8:**
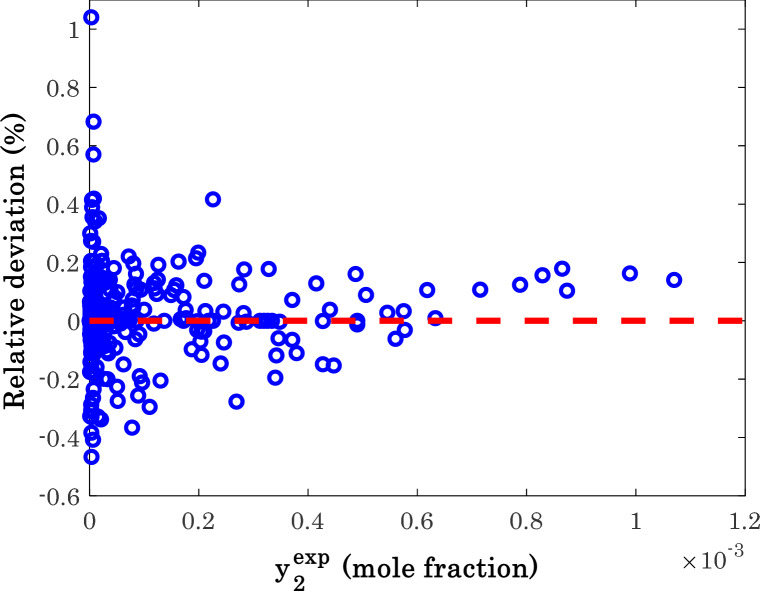
The observed relative deviations for estimating each experimental measurement of anti-cancer drug solubility in supercritical carbon dioxide.

Figure 8 investigates the performance of the modified Arrhenius correlation as a function of its relative deviation (RD) for the available database. Equation (17b) expresses the formulation of the RD index^73^.

17b $$RD = \left( {\frac{{y_{2}^{\exp } - y_{2}^{cal} }}{{y_{2}^{\exp } }}} \right)_{i} \;\;\;i = 1,\;2,\; \ldots ,\;N$$

This figure confirms that the proposed correlation has successfully correlate the experimental solubility data to its corresponding influential variables. Excluding only three experiments, all other solubility measurements are estimated with the − 0.5 < RD < 0.5.

### Differentiating between outlier/valid data

**Figure 9:**
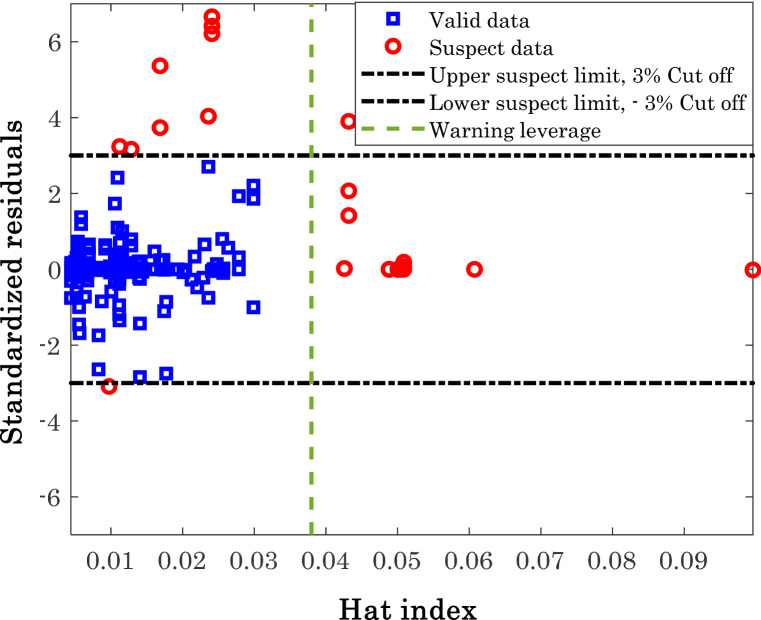
Differentiating between valid and suspect data collected from the literature.

The focus of this section is concentrated on diagnosis of either valid and suspect data. The experimentally-measured information often contain noises^74^ and uncertainties^75^. The leverage method is used to conduct this analysis^76^. As Fig. 9 shows, the leverage method discriminates between the valid (□ symbols) and suspect (○ symbols) information by plotting the standardized residual (SR) as a function of hat index. The SR can be obtained by dividing the residual error (RE) by its standard deviation (SD). Equations (18) to (21) present the RE, average value of RE, SD, and SR formula, respectively^77,78^.

18 $$RE = \left( {y_{2}^{\exp } - y_{2}^{cal} } \right)_{i} \;\;\;i = 1,\;2,\;...,\;N$$

19 $$\overline{RE} = \frac{1}{N} \times \sum\limits_{i = 1}^{N} {RE_{i} }$$

20 $$SD = \sqrt {\frac{1}{N} \times \sum\limits_{i = 1}^{N} {\left( {RE - \overline{RE} } \right)_{i}^{2} } }$$

21 $$SR = \left( {\frac{RE}{{SD}}} \right)_{i} \;\;\;i = 1,\;2,\; \ldots ,N$$

Applying the leverage method on the experimental databank and estimated values of anti-cancer drug solubility (Fig. 9) justifies that the major segment of the experimental data (92.72%) is valid, and only 23 datasets may be outliers.

The excellent accuracy of the modified Arrhenius correlation is previously approved using experimental data and comparison by other available models in the literature. Moreover, the current analysis confirms the validity of the experimental databank. Therefore, it can be claimed that the modified Arrhenius correlation can be readily used in the real application.

**Figure 10:**
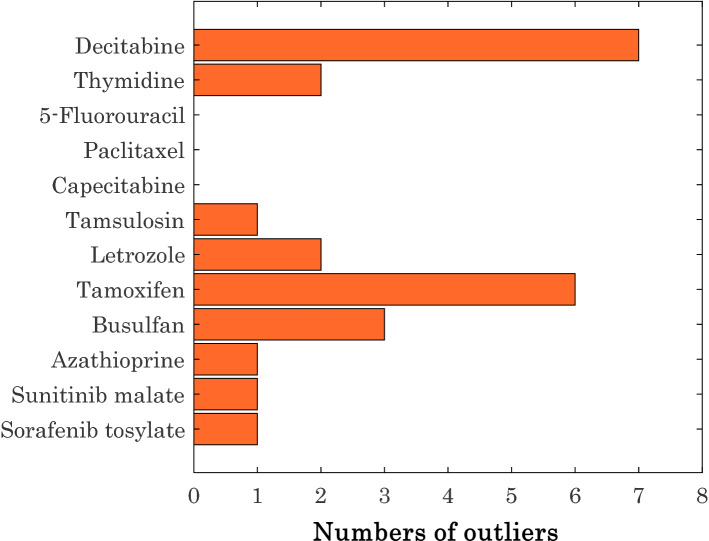
Numbers of detected outliers for the considered anti-cancer drugs.

The numbers of possible outlier for each anti-cancer drug are reported in Fig. 10. It seems that the experimental solubility data for capecitabine, paclitaxel, and 5-fluorouracil with no outlier are the most reliable information. On the other hand, the solubility measurements of decitabine and tamoxifen (with seven and six outliers) in SCCO_2_ are the under-question experiments.

### Investigating the effect of operating conditions

It is previously shown in Table 4 that the modified Arrhenius correlation predict sunitinib malate (AARD = 3.89%) and thymidine (AARD = 16.64%) with the highest and lowest accuracies, respectively. This section investigates the effect of pressure and temperature on the solubility of these anti-cancer drugs in the SCCO_2_ both experimentally and modeling.

**Figure 11:**
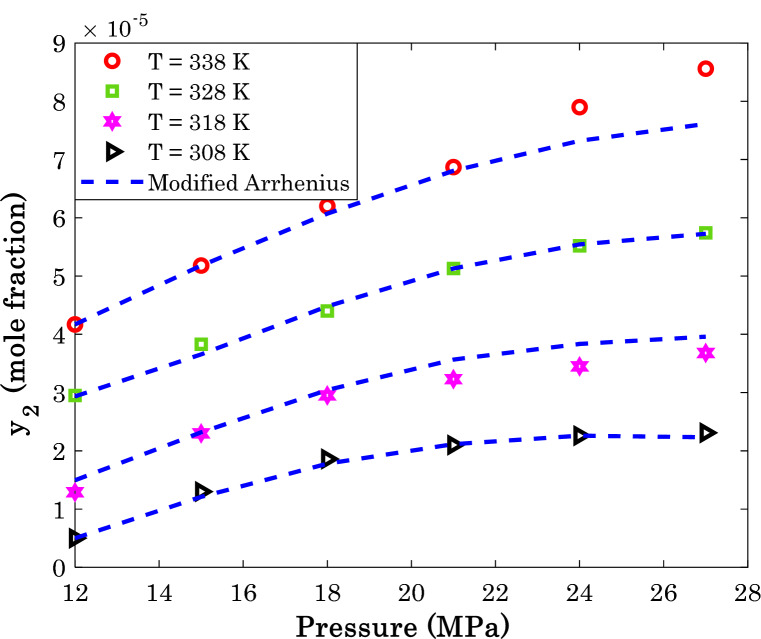
Variation of the sunitinib malate solubility in the supercritical CO_2_ as a function of operating pressure and temperature.

**Figure 12:**
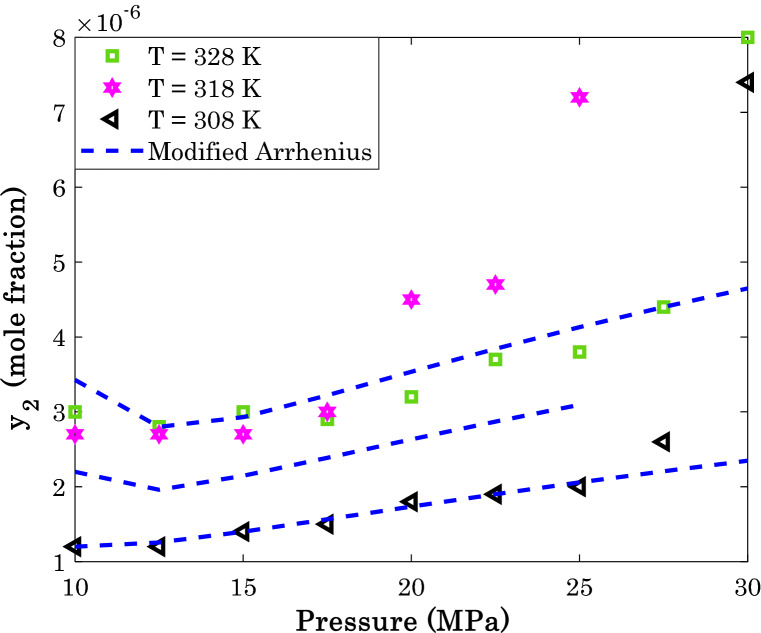
The effect of pressure and temperature on the thymidine solubility in the supercritical carbon dioxide.

Figure 11 explains the effect of isothermal variation of the operating pressure on the sunitinib malate in supercritical carbon dioxide, while Fig. 12 is associated with the thymidine/SCCO_2_ binary system.

Excluding some scattering data in Fig. 12, generally the solubility of anti-cancer drugs in SCCO_2_ increase by increasing either pressure or temperature. This finding is in complete agreement of relevancy analysis (see Fig. 1). Moreover, an acceptable level of agreement exists between actual solubility data and their associated predictions by the modified Arrhenius correlation.

A relatively high scattering measurements for thymidine/SCCO_2_ system (especially at higher temperatures) is responsible for observed deviation between actual and modeling data. It is worth noting that this is the most accurate predictions among eleven different empirical correlations (Supplementary Information).

### Investigating the effect drug type

By measuring the average value of solubility of different anti-cancer drugs, it is concluded that busulfan and tamoxifen have the highest tendency for dissolution in supercritical CO_2_, while the sorafenib tosylate and tamsulosin show the lowest tendency.

**Figure 13:**
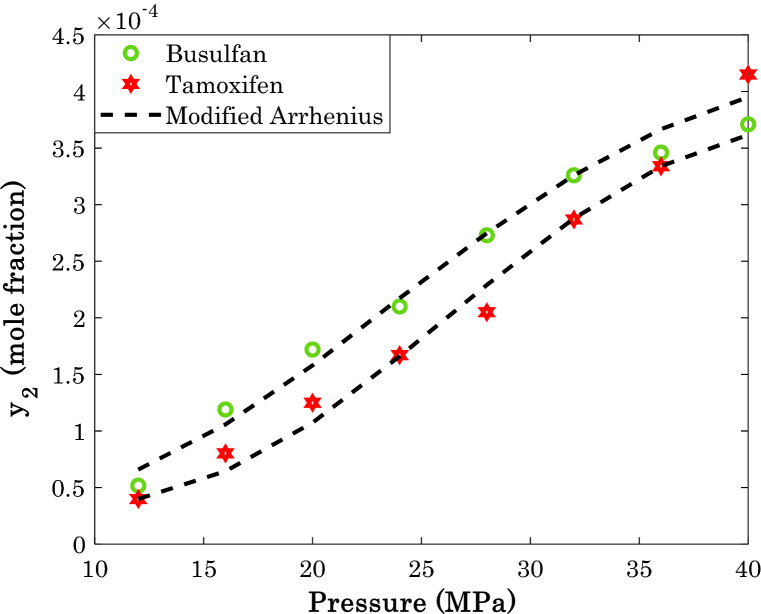
The highest amount of drug solubility in SCCO_2_ at temperature = 318 K.

**Figure 14:**
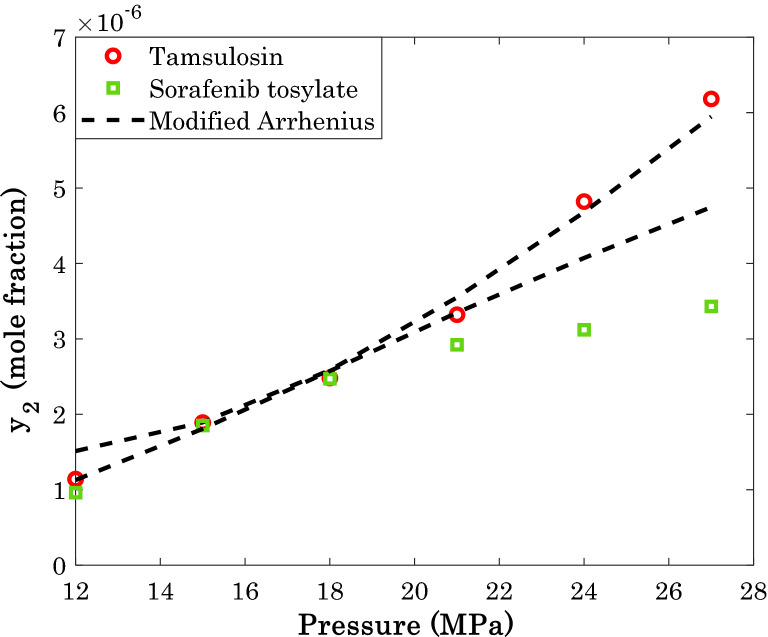
The smallest amount of drug solubility in SCCO_2_ at temperature = 318 K.

Figures 13 and 14 present the modeling and experimental data for two high-soluble and two low-soluble anti-cancer drugs in SCCO_2_, respectively. The provided AARD of 7.92% (busulfan) and 7.40% (tamoxifen) for the high-soluble anti-cancer drugs by the modified Arrhenius correlation is a justification for excellent performance of the model.

On the other hand, the proposed correlation predicts the solubility of the low-soluble anti-cancer drugs with the AARD of 8.44% (tamsulosin) and 17.92% (sorafenib tosylate).

It should be mentioned that this level of uncertainty for this ultra-low variable (anti-cancer drug solubility in SCCO_2_) has its own scientific and real-field merits.

### Maximum achievable drug solubility in SCCO_2_

**Figure 15:**
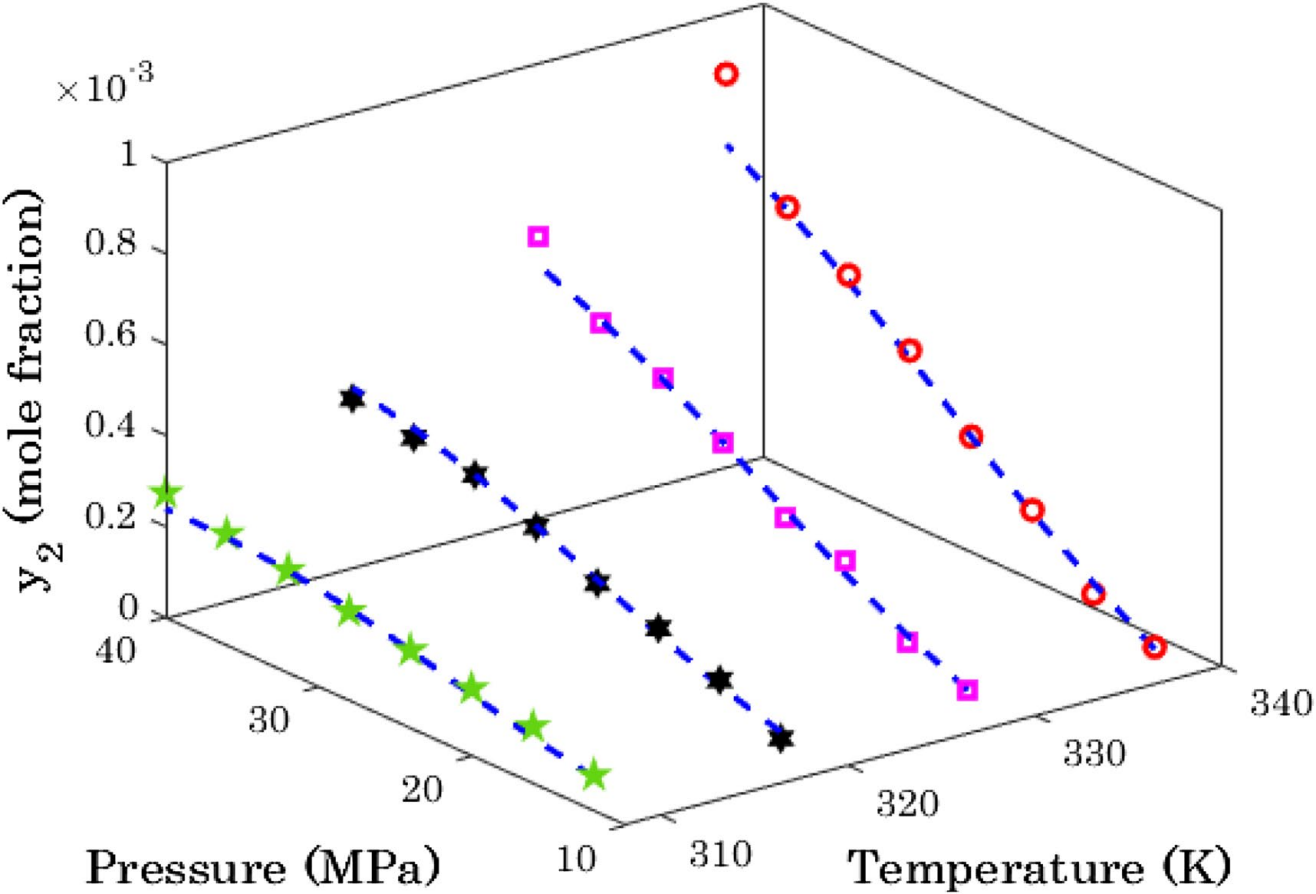
Effect of operating conditions on the busulfan solubility in SCCO_2_ (open circle: 338 K, open rectangle: 328 K, six pointed filled star: 318 K, four pointed filled star: 308 K, dashed lines modified Arrhenius predictions).

The previous analysis approved that the busulfan is the most soluble anti-cancer drug in the supercritical CO_2_. Therefore, for locating the operating condition that maximizes the busulfan solubility in the SCCO_2_, it is necessary to monitor it for all pressures and temperatures. Figure 15 exhibits the busulfan solubility in SCCO_2_ for all possible operating conditions from experimental and modeling perspectives.

Like all other analyses, an excellent performance of the modified Arrhenius correlation can be justified in this analysis too. This figure also clarifies that the positive effect of pressure on the drug solubility intensifies by increasing the temperature. In other word, the slope of solubility with respect to the pressure increases by increasing temperature.

Finally, both experimental data and modeling results show that the highest busulfan solubility in the SCCO_2_ may be achieved at the highest allowable temperature and pressure (i.e., P = 40 bar, T = 338 K).

## Conclusion

A combination of the Arrhenius-shape and departure functions is proposed to correlate the anti-cancer drug solubility in the supercritical carbon dioxide. The pre-exponential part of the Arrhenius-shape term is linearly related to the temperature and carbon dioxide density, and its exponential part inversely relates to the pressure. The departure function is directly related to the natural logarithm of the carbon dioxide density to the temperature ratio. The developed correlation outperformed all well-known literature equations for predicting the solute solubility in supercritical carbon dioxide. The modified Arrhenius correlation provided the AARD = 9.54% and R^2^ = 0.98479 for estimating all experimental datasets in the literature. In contrast, the most accurate correlation in the literature (i.e., Bian et al. correlation) showed the AARD = 14.90% for predicting the considered database. It is possible to improve predicting accuracy of anti-cancer drug solubility in supercritical CO_2_ by more than 56% using the developed correlation in this study. The relevancy analysis exhibited that anti-cancer drug solubility in supercritical CO_2_ increases by increasing either pressure and temperature. Furthermore, it is found that less than 7.5% of the literature data are suspect information, and the remaining 92.5% are valid measurements. The provided Supplementary Material reports the adjusted coefficients of the available empirical correlations in the literature.

## Supplementary Information

Supplementary Information.

## Data Availability

All data generated or analyzed during this study are available on reasonable request from the corresponding author.
