# Supplementary material for: Developing an accurate empirical correlation for predicting anti-cancer drugs’ dissolution in supercritical carbon dioxide
Source: Sci Rep. 2022 Jun 7;12:9380. doi: 10.1038/s41598-022-13233-x (PMC9174250; doi:10.1038/s41598-022-13233-x)
Supplement: Supplementary file 1 — Supplementary Information. [file 41598_2022_13233_MOESM1_ESM.docx]

Adjusted coefficients of the available empirical correlations in the literature

| Anti-cancer drugs | Adjusted coefficients for Eq. (1), i.e., Chrastil [1] | | | Reference |
| --- | --- | --- | --- | --- |
|  | a_1_ | a_2_ | a_3_ |  |
| Sorafenib tosylate | 4.6 | -8886.4 | -6.5938 | [2] |
| Sunitinib malate | 2.9384 | -5999.5353 | -2.0082 | [3] |
| Azathioprine | 3.7654 | -4405.0456 | -14.4169 | [4] |
| Busulfan | 20.263 | -7875.1 | 0.106 | [5] |
| Tamoxifen | -7692.4 | 22.38 | 6.82 | [6] |
| Letrozole | 4.275263436 | -4114.330377 | -17.73837331 | This study |
| Tamsulosin | 6.639 | -29.828 | -2993.637 | [7] |
| Capecitabine | 10.6 | -8509 | -55.3 | [8] |
| Paclitaxel | 1.345408449 | -2788.643504 | -3.512332996 | This study |
| 5-Fluorouracil | 0.448796173 | -1500.58731 | -0.858246254 | This study |
| Thymidine | 1.090834963 | -2766.299088 | -2.135803019 | This study |
| Decitabine | -7402.5 | 23.225 | 6.84 | [9] |

| Anti-cancer drugs | Adjusted coefficients for Eq. (2), i.e., Jouyban et al. [10] | | | | | | Reference |
| --- | --- | --- | --- | --- | --- | --- | --- |
|  | a_1_ | a_2_ | a_3_ | a_4_ | a_5_ | a_6_ |  |
| Sorafenib tosylate | 4 | -23.3 | -0.000053 | 0.0001 | -0.00009 | 13.1524 | [2] |
| Sunitinib malate | 3.4016 | -13.4687 | 0.000003 | 0.000007 | -1.6461 | 4.1346 | [3] |
| Azathioprine | 5.2502 | -16.3613 | 0.0000002 | 0.0000039 | -1.2385 | 8.8510 | [4] |
| Busulfan | 28.25025 | 0.019964938 | -0.198385799 | 0.005241015 | 0.104821862 | -6.651752711 | This study |
| Tamoxifen | 86.58539 | 79.25216462 | -39.24392423 | -15.2203276 | 37.17645754 | 10.64111266 | This study |
| Letrozole | -22.59 | 0.000979 | -0.0000795 | 0.000115 | 0.63 | 1.2 | [11] |
| Tamsulosin | -10.52 | 0.027351747 | -0.793037285 | 0.043914688 | -2.88677289 | 0.702852289 | This study |
| Capecitabine | -32.58 | -0.71 | 0.0000156 | 0 | -1.8 | -3.94 | [8] |
| Paclitaxel | -59.0198 | -0.45455128 | 1.769712812 | -0.10668742 | 24.71610076 | -36.19244462 | This study |
| 5-Fluorouracil | -11.6394 | -0.01357816 | -0.171976918 | 0.00811335 | -1.36361496 | 6.197367321 | This study |
| Thymidine | -153.104 | 0.081212185 | 0.126187599 | -1.26550163 | 133.7548936 | -22.93262271 | This study |
| Decitabine | 70.61937 | 0.031882051 | -0.902036268 | 0.019427588 | 0.002852762 | -6.721909636 | This study |

| Anti-cancer drugs | Adjusted coefficients for Eq. (3), i.e., Kumar and Johnstone [12] | | | Reference |
| --- | --- | --- | --- | --- |
|  | a_1_ | a_2_ | a_3_ |  |
| Sorafenib tosylate | 11.8 | 0.005 | -9126.8266 | [2] |
| Sunitinib malate | 0.615125 | -0.00498 | -2767.19 | This study |
| Azathioprine | -3.77191 | 0.178721 | -2813.43 | This study |
| Busulfan | 3.19 | -5481.5 | 0.29 | [5] |
| Tamoxifen | 8.6 | -7660.6 | 0.35 | [6] |
| Letrozole | -11.5179 | 0.178019 | -283.866 | This study |
| Tamsulosin | -5.144 | 0.387 | -4653.784 | [7] |
| Capecitabine | 4.99 | 0.013 | -8465 | [8] |
| Paclitaxel | -11.5618 | 0.227835 | -1041.26 | This study |
| 5-Fluorouracil | -4.03693 | -0.03466 | -1577.39 | This study |
| Thymidine | -6.09743 | -0.00992 | -1391.89 | This study |
| Decitabine | 7.8 | -7327.4 | 0.348 | [9] |

| Anti-cancer drugs | Adjusted coefficients for Eq. (4), i.e., Garlapati and Madras [13] | | | | | Reference |
| --- | --- | --- | --- | --- | --- | --- |
|  | a_1_ | a_2_ | a_3_ | a_4_ | a_5_ |  |
| Sorafenib tosylate | -135.8 | -26.2 | 0.0011 | -1872.2054 | 0.0239 | [2] |
| Sunitinib malate | -61.7257 | -15.8357 | 0.0009 | -2627.0077 | 12.8562 | [3] |
| Azathioprine | -1051.7082 | -138.4507 | 0.0047 | -3257.3941 | 148.3136 | [4] |
| Busulfan | Not reported | Not reported | Not reported | Not reported | Not reported | [5] |
| Tamoxifen | -63.07 | 0 | 0 | -5810 | 5.8 | [6] |
| Letrozole | Not reported | Not reported | Not reported | Not reported | Not reported | [11] |
| Tamsulosin | -43.217 | 5.165762 | -0.00034 | 12.63038 | -0.00172 | This study |
| Capecitabine | -9.99957 | -36.4249 | 0.004706 | 40.26143 | 17.59794 | This study |
| Paclitaxel | -12.9991 | -48.135 | 0.004252 | 462.9006 | 24.11079 | This study |
| 5-Fluorouracil | 8.949875 | -12.2271 | 0.001614 | -172.601 | 4.418246 | This study |
| Thymidine | -11.1598 | -3.66683 | 0.000271 | -592.225 | 2.038211 | This study |
| Decitabine | -64.05 | 0 | 0 | -5513.7 | 5.83 | [9] |

| Anti-cancer drugs | Adjusted coefficients for Eq. (5), i.e., Bian et al. [14] | | | | | Reference |
| --- | --- | --- | --- | --- | --- | --- |
|  | a_1_ | a_2_ | a_3_ | a_4_ | a_5_ |  |
| Sorafenib tosylate | -1.5 | 0.005 | -1284.3150 | -9.7635 | -0.0006 | [2] |
| Sunitinib malate | -1.2018 | 0.0034 | -1234.4274 | -6.9288 | 09095 | [3] |
| Azathioprine | 0.1917 | -0.0005 | -6835.9120 | 2.5403 | 4.7293 | [4] |
| Busulfan | -0.85731 | 0.003777 | 13.50644 | -6.78282 | -3.88514 | This study |
| Tamoxifen | -0.73087 | 0.005171 | 293.7783 | -9.83579 | -5.73639 | This study |
| Letrozole | 3.352831 | 0.003936 | -453.386 | -9.43201 | -27.6665 | This study |
| Tamsulosin | -31.572 | -10.890 | 3849.022 | 0.777 | 0.005 | [7] |
| Capecitabine |  |  |  |  |  | This study |
| Paclitaxel | 0.79001 | 0.003225 | 6.091919 | -8.19228 | -12.285 | This study |
| 5-Fluorouracil | 6.099083 | 0.001552 | 7.07048 | -5.6407 | -44.3716 | This study |
| Thymidine | -2.86771 | 0.003359 | 19.78307 | -6.07904 | 5.821965 | This study |
| Decitabine | 1.049436 | 0.003986 | -1102.63 | -7.55414 | -12.4352 | This study |

| Anti-cancer drugs | Adjusted coefficients for Eq. (6), i.e., Bartle et al. [15] | | | Reference |
| --- | --- | --- | --- | --- |
|  | a_1_ | a_2_ | a_3_ |  |
| Sorafenib tosylate | 26.1 | -11006.4 | 0.0086 | [2] |
| Sunitinib malate | 22.9194 | -9208.3268 | 0.0083 | [3] |
| Azathioprine | 13.8694 | -6669.2033 | 0.0071 | [4] |
| Busulfan | 20.263 | -7875.1 | 0.106 | [5] |
| Tamoxifen | 26.604 | -10046 | 0.01195 | [6] |
| Letrozole | -1.70265 | -815.351 | 0.00592 | This study |
| Tamsulosin | 9.468 | 0.0105 | -5707.259 | [7] |
| Capecitabine | -4.42438 | 6.074035 | 0.007837 | This study |
| Paclitaxel | -3.48548 | -1008.47 | 0.008796 | This study |
| 5-Fluorouracil | -1.79639 | -859.924 | 0.000476 | This study |
| Thymidine | -3.17846 | -785.896 | 0.000626 | This study |
| Decitabine | 25.758 | -9710.2 | 0.0119 | [9] |

| Anti-cancer drugs | Adjusted coefficients for Eq. (7), i.e., Méndez-Santiago and Teja [16] | | | Reference |
| --- | --- | --- | --- | --- |
|  | a_1_ | a_2_ | a_3_ |  |
| Sorafenib tosylate | -13825.4 | 130844.2 | 28.3276 | [2] |
| Sunitinib malate | -11163.0201 | 115756.6570 | 23.2764 | [3] |
| Azathioprine | -8561.5101 | 103530.4602 | 14.6113 | [4] |
| Busulfan | -10635 | 21.399 | 3.41 | [5] |
| Tamoxifen | -13158 | 27.87 | 3.85 | [6] |
| Letrozole | 11369 | 3.11 | 29.01 | [11] |
| Tamsulosin | -9604.557 | 3.820 | 13.111 | [7] |
| Capecitabine | -14569 | 5.304 | 26.537 | [8] |
| Paclitaxel | -1062.08 | 2.709098 | -11.7711 | This study |
| 5-Fluorouracil | -2764.52 | -0.01734 | 1.999905 | This study |
| Thymidine | -2556.94 | 0.440563 | -0.86497 | This study |
| Decitabine | -12862 | 27.145 | 3.825 | [9] |

| Anti-cancer drugs | Adjusted coefficients for Eq. (8), i.e., Sodeifian et al. [17] | | | | | | Reference |
| --- | --- | --- | --- | --- | --- | --- | --- |
|  | a_1_ | a_2_ | a_3_ | a_4_ | a_5_ | a_6_ |  |
| Sorafenib tosylate | -100.3 | -1.7 | 7.533 | -0.0016 | 0.05 | -84.6830 | [2] |
| Sunitinib malate | 1.4844 | -1.5762 | -0.4674 | 0.0004 | 0.0441 | -528.0951 | [3] |
| Azathioprine | -9.3294 | -0.1039 | 0.7221 | 0.0011 | -0.0005 | -807.1890 | [4] |
| Busulfan | -2.69848 | 0.391382 | -0.0239 | 0.000859 | -0.00878 | -365.492 | This study |
| Tamoxifen | -12.4364 | -5.30633 | -1.74249 | 0.001054 | 0.19974 | 106.4259 | This study |
| Letrozole | -9.3145 | -2.77972 | -0.26657 | 0.000158 | 0.090868 | -218.496 | This study |
| Tamsulosin | -51.217 | 1.773 | 4.252 | 0.001 | -0.042 | 823.603 | [7] |
| Capecitabine | -12.6988 | -5.13509 | -1.28361 | 0.001339 | 0.159351 | 4.920852 | This study |
| Paclitaxel | 134.5283 | -2.4308 | -12.3093 | 0.000332 | 0.085278 | -1.47547 | This study |
| 5-Fluorouracil | 66.93184 | -1.83051 | -6.64774 | 0.000379 | 0.05714 | 3.104433 | This study |
| Thymidine | -10.0552 | -1.98483 | -0.19089 | -0.0004 | 0.055502 | 8.681183 | This study |
| Decitabine | -11.1638 | 4.696832 | 0.838015 | -0.00019 | -0.12713 | 31.84026 | This study |

| Anti-cancer drugs | Adjusted coefficients for Eq. (9), i.e., Tan et al. [18] | | | | Reference |
| --- | --- | --- | --- | --- | --- |
|  | a_1_ | a_2_ | a_3_ | a_4_ |  |
| Sorafenib tosylate | -0.34153 | 0.000164 | 12.16276 | -7.0818 | This study |
| Sunitinib malate | 0.332106 | -0.00068 | 19.93995 | -12.0404 | This study |
| Azathioprine | 12.15759 | -0.01329 | 18.98903 | -150.049 | This study |
| Busulfan | 2.81136 | 0.000131 | 19.61218 | -41.5707 | This study |
| Tamoxifen | -0.02559 | 0.004046 | -315.446 | -8.89421 | This study |
| Letrozole | 0.18177 | 0.003758 | -295.654 | -13.5041 | This study |
| Tamsulosin | -0.3347 | 0.005427 | 1.634496 | -10.7356 | This study |
| Capecitabine | 2.827 | 0.008 | -7260 | -30.3 | [8] |
| Paclitaxel | 20.17869 | -0.02523 | 1.756306 | -241.327 | This study |
| 5-Fluorouracil | -0.00197 | -0.00096 | 7.954322 | -8.87566 | This study |
| Thymidine | 3.168417 | -0.0053 | 8.104563 | -45.8643 | This study |
| Decitabine | -0.0143 | 0.006108 | -7.2038 | -11.5945 | This study |

| Anti-cancer drugs | Adjusted coefficients for Eq. (10), i.e., Gordillo et al. [19] | | | | | | Reference |
| --- | --- | --- | --- | --- | --- | --- | --- |
|  | a_1_ | a_2_ | a_3_ | a_4_ | a_5_ | a_6_ |  |
| Sorafenib tosylate | 1.147165 | 5.602838 | 29.31159 | 54.3577 | 16.09496 | -5.04435 | This study |
| Sunitinib malate | -12.3186 | -0.16624 | -1.24198 | 0.099917 | -1.20084 | 0.001628 | This study |
| Azathioprine | -8.08907 | 29.89165 | -3.24859 | -4.92077 | 59.7211 | -0.00105 | This study |
| Busulfan | 15.66554 | -3.0934 | 43.96169 | 67.12683 | -21.9812 | -9.38679 | This study |
| Tamoxifen | 1.477635 | 12.04623 | 68.41506 | 24.49421 | -35.9844 | -4.2233 | This study |
| Letrozole | 141.7499 | -11.1352 | 47.42107 | -2.03074 | -75.4173 | -0.13775 | This study |
| Tamsulosin | 181.5051 | 57.21996 | -40.5689 | 6.731101 | -3.08679 | -0.28659 | This study |
| Capecitabine | -24.47 | -0.086 | -0.0000916 | 1.6× 10^-10^ | 0.14 | 1.0× 10^-10^ | [8] |
| Paclitaxel | -11.3499 | -4.58843 | -2.01892 | 86.20906 | -17.5232 | -7.62291 | This study |
| 5-Fluorouracil | -24.9314 | 29.62369 | 15.66656 | -2.00347 | 7.351673 | 0.027888 | This study |
| Thymidine | 29.52988 | 18.50951 | 71.73559 | 128.6456 | -10.283 | -13.1839 | This study |
| Decitabine | -17.5257 | -0.23538 | 1.845616 | 23.80618 | -83.342 | -2.85203 | This study |

**References**

[1] K. Aim, M. Fermeglia, Solubility of solids and liquids in supercritical fluids, Exp. Determ. Solubilities. 86 (2005) 491–555.

[2] G. Sodeifian, F. Razmimanesh, S.A. Sajadian, Prediction of solubility of sunitinib malate (an anti-cancer drug) in supercritical carbon dioxide (SC–CO_2_): Experimental correlations and thermodynamic modeling, J. Mol. Liq. 297 (2020) 105998.

[3] G. Sodeifian, N. Saadati Ardestani, S.A. Sajadian, M.R. Golmohammadi, A. Fazlali, Prediction of solubility of sodium valproate in supercritical carbon dioxide: Experimental study and thermodynamic modeling, ACS Appl. Mater. Interfaces. 297 (2020) 111740.

[4] G. Sodeifian, F. Razmimanesh, N. Saadati Ardestani, S.A. Sajadian, Experimental data and thermodynamic modeling of solubility of Azathioprine, as an immunosuppressive and anti-cancer drug, in supercritical carbon dioxide, J. Mol. Liq. 299 (2020) 112179.

[5] M. Pishnamazi, S. Zabihi, S. Jamshidian, H.Z. Hezaveh, A.Z. Hezave, S. Shirazian, Measuring solubility of a chemotherapy-anti cancer drug (busulfan) in supercritical carbon dioxide, J. Mol. Liq. 317 (2020) 113954.

[6] M. Pishnamazi, S. Zabihi, S. Jamshidian, F. Borousan, A.Z. Hezave, S. Shirazian, Thermodynamic modelling and experimental validation of pharmaceutical solubility in supercritical solvent, J. Mol. Liq. 319 (2020) 114120.

[7] S.M. Hazaveie, G. Sodeifian, S.A. Sajadian, Measurement and thermodynamic modeling of solubility of Tamsulosin drug (anti cancer and anti-prostatic tumor activity) in supercritical carbon dioxide, J. Supercrit. Fluids. 163 (2020) 104875.

[8] Y. Yamini, M. Hojjati, P. Kalantarian, M. Moradi, A. Esrafili, A. Vatanara, Solubility of capecitabine and docetaxel in supercritical carbon dioxide: Data and the best correlation, Thermochim. Acta. 549 (2012) 95–101.

[9] M. Pishnamazi, S. Zabihi, S. Jamshidian, F. Borousan, A.Z. Hezave, A. Marjani, S. Shirazian, Experimental and thermodynamic modeling decitabine anti cancer drug solubility in supercritical carbon dioxide, Sci. Rep. 11 (2021) 1–8.

[10] A. Jouyban, M. Rehman, B.Y. Shekunov, H.K. Chan, B.J. Clark, P. York, Solubility prediction in supercritical CO_2_ using minimum number of experiments, J. Pharm. Sci. 91 (2002) 1287–1295.

[11] G. Sodeifian, S.A. Sajadian, Solubility measurement and preparation of nanoparticles of an anticancer drug (Letrozole) using rapid expansion of supercritical solutions with solid cosolvent (RESS-SC), J. Supercrit. Fluids. 133 (2018) 239–252.

[12] S.K. Kumar, K.P. Johnston, Modelling the solubility of solids in supercritical fluids with density as the independent variable, J. Supercrit. Fluids. 1 (1988) 15–22.

[13] C. Garlapati, G. Madras, New empirical expressions to correlate solubilities of solids in supercritical carbon dioxide, Thermochim. Acta. 500 (2010) 123–127.

[14] X.Q. Bian, Q. Zhang, Z.M. Du, J. Chen, J.N. Jaubert, A five-parameter empirical model for correlating the solubility of solid compounds in supercritical carbon dioxide, Fluid Phase Equilib. 411 (2016) 74–80.

[15] K.D. Bartle, A.A. Clifford, S.A. Jafar, G.F. Shilstone, Solubilities of solids and liquids of low volatility in supercritical carbon dioxide, J. Phys. Chem. Ref. Data. 20 (1991) 713–756.

[16] J. Méndez-Santiago, A.S. Teja, The solubility of solids in supercritical fluids, Fluid Phase Equilib. 158–160 (1999) 501–510.

[17] G. Sodeifian, F. Razmimanesh, S.A. Sajadian, Solubility measurement of a chemotherapeutic agent (Imatinib mesylate) in supercritical carbon dioxide: Assessment of new empirical model, J. Supercrit. Fluids. 146 (2019) 89–99.

[18] T. Fei, Y. Jichu, S. Hongyao, W. Jiading, Study on the solubility of substances in supercritical fluids, J. Chem. Ind. Eng. 4 (1989) 402–409.

[19] M.D. Gordillo, M.A. Blanco, A. Molero, E. Martinez De La Ossa, Solubility of the antibiotic Penicillin G in supercritical carbon dioxide, J. Supercrit. Fluids. 15 (1999) 183–190.
